# Sustainment of diverse evidence-informed practices disseminated in the Veterans Health Administration (VHA): initial development and piloting of a pragmatic survey tool

**DOI:** 10.1186/s43058-022-00386-z

**Published:** 2023-01-16

**Authors:** Caitlin M. Reardon, Laura Damschroder, Marilla A. Opra Widerquist, Maria Arasim, George L. Jackson, Brandolyn White, Sarah L. Cutrona, Gemmae M. Fix, Allen L. Gifford, Kathryn DeLaughter, Heather A. King, Blake Henderson, Ryan Vega, Andrea L. Nevedal

**Affiliations:** 1 Center for Clinical Management Research (CCMR), VA Ann Arbor Healthcare System, Ann Arbor, USA; 2 Center of Innovation to Accelerate Discovery and Practice Transformation (ADAPT), Durham VA Health Care System, Durham, USA; 3 Department of Population Health Sciences, Duke University, Durham, USA; 4 Division of General Internal Medicine, Duke University, Durham, USA; 5 Department of Family Medicine & Community Health, Duke University, Durham, USA; 6 Center for Healthcare Organization & Implementation Research (CHOIR), Bedford & Boston VA Medical Centers, Bedford, USA; 7 Department of Population and Quantitative Health Sciences, UMass Chan Medical School, Worcester, USA; 8 Division of General Internal Medicine, University of Massachusetts Medical School, Worcester, USA; 9 Section of General Internal Medicine, Boston University School of Medicine, Boston, USA; 10 Department of Health Law, Policy & Management, Boston University, Boston, USA; 11 Innovation Ecosystem, United States Veterans Health Administration, Washington, D.C., USA

**Keywords:** Sustainability, Sustainment, Measurement, Outcomes, Model of diffusion, Consolidated Framework for Implementation Research (CFIR)

## Abstract

**Background:**

There are challenges associated with measuring sustainment of evidence-informed practices (EIPs). First, the terms sustainability and sustainment are often falsely conflated: sustainability assesses the likelihood of an EIP being in use in the future while sustainment assesses the extent to which an EIP is (or is not) in use. Second, grant funding often ends before sustainment can be assessed.

The Veterans Health Administration (VHA) Diffusion of Excellence (DoE) program is one of few large-scale models of diffusion; it seeks to identify and disseminate practices across the VHA system. The DoE sponsors “Shark Tank” competitions, in which leaders bid on the opportunity to implement a practice with approximately 6 months of implementation support. As part of an ongoing evaluation of the DoE, we sought to develop and pilot a pragmatic survey tool to assess sustainment of DoE practices.

**Methods:**

In June 2020, surveys were sent to 64 facilities that were part of the DoE evaluation. We began analysis by comparing alignment of quantitative and qualitative responses; some facility representatives reported in the open-text box of the survey that their practice was on a temporary hold due to COVID-19 but answered the primary outcome question differently. As a result, the team reclassified the primary outcome of these facilities to Sustained: Temporary COVID-Hold. Following this reclassification, the number and percent of facilities in each category was calculated. We used directed content analysis, guided by the Consolidated Framework for Implementation Research (CFIR), to analyze open-text box responses.

**Results:**

A representative from forty-one facilities (64%) completed the survey. Among responding facilities, 29/41 sustained their practice, 1/41 partially sustained their practice, 8/41 had not sustained their practice, and 3/41 had never implemented their practice. Sustainment rates increased between Cohorts 1–4.

**Conclusions:**

The initial development and piloting of our pragmatic survey allowed us to assess sustainment of DoE practices. Planned updates to the survey will enable flexibility in assessing sustainment and its determinants at any phase after adoption. This assessment approach can flex with the longitudinal and dynamic nature of sustainment, including capturing nuances in outcomes when practices are on a temporary hold. If additional piloting illustrates the survey is useful, we plan to assess the reliability and validity of this measure for broader use in the field.

**Supplementary Information:**

The online version contains supplementary material available at 10.1186/s43058-022-00386-z.

Contributions to the literature • The terms sustainability and sustainment are used interchangeably in the literature; this paper provides clarity in defining and differentiating these terms.

• Sustainment determinants and outcomes are often conflated in the literature; this paper illustrates that many sustainment determinants are inaccurately described as outcomes.

• Sustainment is dynamic; this paper provides an approach to better capture nuance in sustainment outcomes when practices are on a temporary hold.

• A high rate of practice sustainment among responding facilities suggests that the VHA DoE is a promising large-scale model of diffusion.

## Background

### Evaluating sustainment of evidence-informed practices is challenging

There is growing interest in sustainment of evidence-informed practices (EIPs) [1, 2]; however, the literature on how to best measure sustainment over time is still developing [3]. Understanding sustainment of EIPs is challenging, which Birken et al. suggest is due to a lack of conceptual clarity and methodological challenges [4].

First, the terms sustainability and sustainment are often used interchangeably [4]. While these terms are related, there are important distinctions. Sustainability assesses the likelihood of an EIP being in use at a future point in time; it is measured by assessing contextual *determinants* (i.e., factors which decisively affect the nature or outcome of something) [5]. For example, the EIP is perceived to have low sustainability due to inadequate funding or lack of priority. Operationally, the goal is to determine whether the conditions indicative of sustaining EIPs are in place, and if not, to guide efforts to put such conditions into place [6, 7].

In contrast, sustainment assesses the extent to which an EIP is (or is not) in use after a specific period of time after initial implementation; for example, the RE-AIM Framework specifies that the sustainment period begins at least 6 months after initial implementation is completed [8]. Sustainment is measured by assessing *outcomes* (i.e., the way a thing turns out; a consequence), e.g., the EIP is in use/not in use. Operationally, the goal is to determine if EIPs are still in place following the end of implementation support [9]. Distinguishing between sustainability and sustainment will help researchers develop shared language and advance implementation science [4, 10].

Second, grant funding periods often end after implementation is completed, so initial and long-term sustainment cannot be assessed due to time and resource constraints [4]. As a result, most measure development has focused on sustainability (which can be measured at any point in time during grant funding periods) not sustainment (which cannot be assessed until after a sufficient amount of time has elapsed) [11]. For example, systematic reviews have highlighted factors influencing sustainability [12–14], and the Program Sustainability Assessment Tool (PSAT) [15] and Program Sustainability Index [16] are frequently used instruments to assess sustainability of innovations, but do not include measures for sustainment.

### Limitations to current sustainment instruments

The existing literature conceptualizes a mix of items as sustainment outcomes, including the presence or absence of an EIP after implementation is completed, such as the continued use of the EIP (and its core components) [11, 17–19] or the level of institutionalization of the EIP [18, 19]. In addition, the literature discusses continued “attention to the issue or problem” addressed by the EIP, even when the specific EIP is no longer in use or is replaced by something else, as a sustainment outcome [18]. Finally, there are several outcomes referenced in the literature that have been used to measure both sustainability and sustainment, such as continued institutional support [11, 17–19] and continued funding for the EIP [11], as well as the continued benefit of the EIP [17–19] (see Table 1). In effect, there is overlap in the literature between sustainment determinants and sustainment outcomes, which increases confusion and hinders advancement in the field. Finally, most instruments do not include open-text boxes, which are an important “resource for improving quantitative measurement accuracy and qualitatively uncovering unexpected responses” [20].

**Table 1:** Survey questions mapped to published sustainment outcomes

Sustainment items	Survey question	Palinkas [11]	Lennox [17]	Scheirer Dearing [18]	Shelton [19]
Primary outcomes					
1. Practice sustainment	Is this practice still being used or done at your site? (yes/no/partially)	X	X	X	X
Secondary outcomes					
2. Practice institutionalization	Is this practice considered routine, usual practice? (i.e., practice is nearly always used or done when appropriate by all individuals involved) (yes/no/partially)			X	X
3. Practice priority	This practice has priority at your site. (strongly disagree to strongly agree Likert scale)			X^a^	
4. Practice buy-in/capacity/partnership^b^	This practice has support and commitment from facility leadership. This practice has a Champion (leader) at your site. This practice has sufficient staffing. This practice has support and buy-in from key outside community entities. (strongly disagree to strongly agree Likert scale)	X	X	X	X
5. Practice funding^b^	This practice has sufficient funding. This practice has sufficient resources (e.g., space, equipment). (strongly disagree to strongly agree Likert scale)	X			
6. Practice benefit^b^	Is this practice demonstrating effectiveness at your site? (yes/no/partially)		X	X	X
7. Practice improvements/adaptation	Have there been any changes or adaptations to this practice? (yes/No/partially)		X		
8. Practice spread/diffusion	Has this practice spread to other units or places in your site? (yes/no/partially)			X	

^a^ Scheirer and Dearing conceptualized this measure as issue priority, not practice priority; however, given that DoE practices assessed different issues, this was presented as practice priority [9]

^b^ A similar item was conceptualized as a sustainment determinant by one or more authors represented in the table [8–10]

Although existing literature offers a variety of single-item sustainment measures for researchers to use, there are few complete pragmatic multi-item instruments. A narrative review by Moullin et al. identified 13 instruments for measuring sustainment. However, they highlighted the need for more pragmatic approaches since many of the existing multi-item sustainment instruments were “overly intervention or context specific” and “lengthy and/or complex” [21]. For example, the Stages of Implementation Completion (SIC) is innovation specific [22] while the Sustainment Measurement System Scale (SMSS) contains 35 items [23]. Furthermore, most multi-item instruments were not well-suited for frontline employees to complete; they were more suited for individuals with expertise in implementation science frameworks [21]. Pragmatic instruments are needed to increase the likelihood participants will understand and respond to all items, especially when it is difficult to incentivize participants over time.

### Organizational context and role of the authors

Our team is embedded within and employed by the United States (US) Veterans Health Administration (VHA), the largest integrated healthcare system in the US. VHA has over 1000 medical centers and community-based outpatient clinics; more information on VHA can be found at www.va.gov.

The VHA Diffusion of Excellence (DoE) is one of few large-scale models of diffusion; it seeks to identify and disseminate EIPs across the VHA system. DoE practices include innovations supported by evidence from research studies and administrative or clinical experience [24, 25] that strive to address patient, employee, and/or facility needs. The DoE sponsors “Shark Tank” competitions, in which regional and facility leaders bid on the opportunity to implement a practice with approximately 6 months of non-monetary external implementation support. Over 1,500 practices were submitted for consideration between Cohorts 1 and 4 (2016–2019) of Shark Tank; the DoE designated 45 as Promising Practices and these were adopted at 64 facilities (some practices were adopted by more than one facility). For additional detail on the VHA, the DoE, and promising practices, see Additional file 1 as well as previous publications [9, 26–28].

Our team was selected in 2016 to conduct an external evaluation of the DoE, to guide program improvements and assess the impact of the program on VHA (see previous publications and Additional file 1 for more information about the evaluation [9, 27–29]). In earlier phases of our evaluation, we focused on implementation and initial sustainment of DoE practices [28]. In brief, we conducted interviews after the 6-month external implementation support period to understand the level of implementation success as well as barriers and facilitators to implementation at the facilities [28]. Participants described a high level of successful implementation after the initial 6-month period of support. Due to extensive external implementation support, facilities were able to complete implementation unless significant barriers related to “centralized decision making, staffing, or resources” delayed implementation [28]. We then evaluated the initial sustainment of these practices by asking facilities to complete follow-up surveys (on average 1.5 years after external support ended). Over 70% of the initially *successful* teams reported their practice was still being used at their facility. Additionally, over 50% of the initially *unsuccessful* teams reported they had since completed implementation and their practice was still being used at their facility [28]. Although some of these initially unsuccessful facilities implemented their practice after external support ended, research suggests that many EIPs are not sustained once implementation support has ceased [30]. As a result, we shifted our focus to the evaluation of ongoing sustainment of DoE practices. The objective of this manuscript is to (1) describe the initial development and piloting of a pragmatic sustainment survey tool and (2) present results on ongoing practice sustainment.

## Methods

### Survey development

**Fig. 1:**
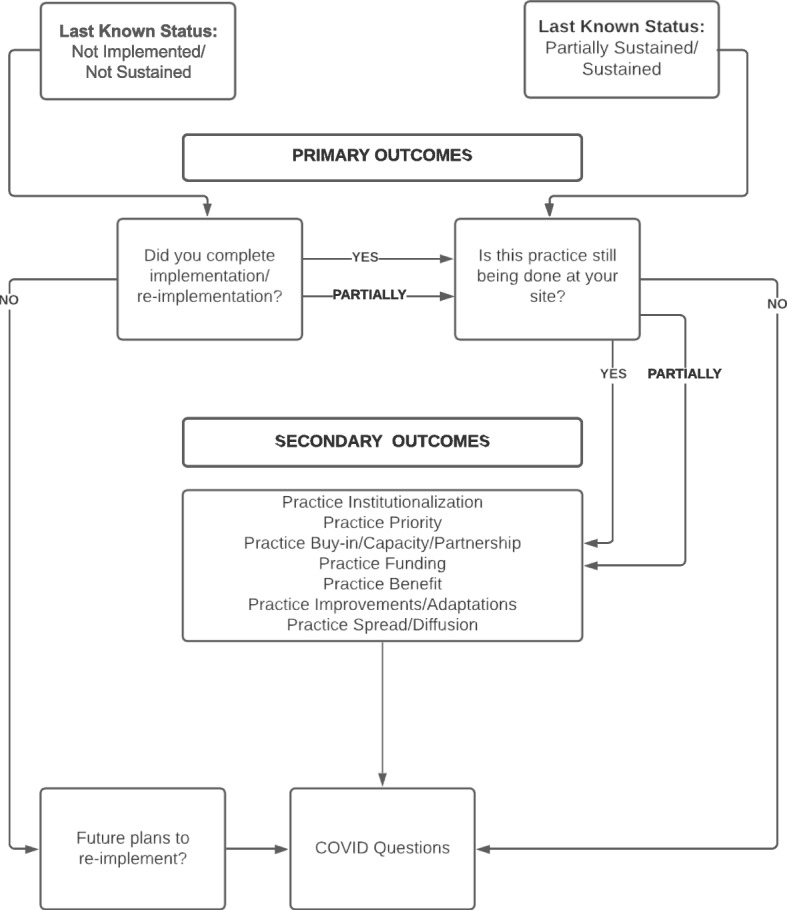
Survey branching logic

To assess the ongoing sustainment of DoE practices, we sought to develop a pragmatic survey that was (1) easy to understand for those without implementation science expertise (i.e., simple), (2) quick to complete (i.e., less than 10 min), and (3) appropriate for 45 different practices (i.e., generic) [21, 31]. Our primary evaluation question for the survey was: Is there ongoing sustainment of DoE practices? To assess this question, we used the last known status of a facility (based on the last interview or survey completed) and branching logic to route respondents through the survey based on their individual facility’s situation (see Fig. 1). Based on our working definition of sustainment, items were conceptualized as primary or secondary outcomes; secondary items were derived from the literature to enhance the survey and provide additional contextual information (see below and Table 1). Furthermore, “Please Describe” open-text boxes were included following all questions so participants could provide additional detail. Descriptions of each outcome are briefly described below; see Table 1 for outcomes mapped to the literature and Additional file 2 for the complete survey.

#### Terms and definitions

##### Primary outcome

As described earlier, our primary outcome is used as the overarching benchmark to determine if a DoE practice is sustained.

- Practice sustainment: extent to which the DoE practice and its core components and activities are in use [11, 17–19]

##### Secondary outcomes

Given the importance of assessing more than whether the practice was in use, the survey included several items from the literature as secondary outcomes. These secondary outcomes provide additional information on the current status of the practice.

- Institutionalization: extent to which the DoE practice is part of routine care and work processes [18, 19]
- Priority: extent to which there is attention to the issue or problem addressed by the DoE practice, i.e., “heightened issue salience” [18]
- Buy-in/capacity/partnership: extent to which key stakeholders and partners support the DoE practice [11, 17–19]
- Funding: extent to which funding is provided to support the DoE practice [11]
- Benefit: extent to which the DoE practice is having the intended outcomes [17–19]
- Improvements/adaptation: extent to which the DoE practice is being improved and/or adapted [17]
- Spread/diffusion: extent to which the DoE practice is spreading or diffusing to other locations [18]

##### Additional survey questions

To assess the fluid and longitudinal nature of sustainment, if a respondent answered “No” to the primary outcome, i.e., the DoE practice was not in use (see above and Table 1) they were asked about future plans to re-implement. If a facility’s representative reported they planned to re-implement their DoE practice, they were retained in the sample for future sustainment surveys. In addition, due to the timing of the sustainment survey (only a few months after the Centers for Disease Control and Prevention (CDC) issued guidance to cancel and/or reschedule non-essential clinical activities) [32], it included questions about the pandemic (see Additional file 2).

##### Data collection

In June 2020, surveys were emailed to representatives of the 64 facilities in Cohorts 1 – 4 that adopted one of the 45 DoE Promising Practices. See Additional file 1 for practice descriptions. Survey follow-up periods ranged from 1 to 3 years, depending on the cohort (i.e., when the practice was adopted). Incentives were not provided to VHA employees because surveys were expected to be completed during working hours. The survey was piloted using the REDCap® platform. Per regulations outlined in VHA Program Guide 1200.21, this evaluation has been designated a non-research quality improvement activity.

##### Data analysis

We calculated the overall response rate and used descriptive statistics (number, percent) to summarize the multiple choice and Likert scale questions. We used directed content analysis, guided by the Consolidated Framework for Implementation Research (CFIR), to analyze open-text box responses [33]. The CFIR is a determinant framework that defines constructs across five domains of potential influences on adoption, implementation, and sustainment [5]: (1) characteristics of the intervention (e.g., evidence strength and quality), (2) outer setting (e.g., patient needs and resources), (3) inner setting (e.g., tension for change), (4) characteristics of Individuals (e.g., self-efficacy), and (5) process (e.g., planning). As one of the most widely cited determinant frameworks, the CFIR was selected to guide analysis in order to facilitate the comparison and translation of our results with other projects. The codebook included deductive CFIR constructs as well as new inductive codes and domains that arose in the data, including relationships between constructs [34]. We used relationship coding to provide a high-level overview of how different constructs interact or relate to each other. See Table 2 for an excerpt of our CFIR informed codebook. Using a consensus-based process [34], two evaluators (CR, AN) coded qualitative data from the open-text boxes and discussed to resolve discrepancies.

**Table 2:** Codebook for open-text responses to survey questions

CFIR construct codes	Operationalized definitions
Characteristics of the innovation domain	Codes capturing information specific to the practice regardless of where it is being implemented
^a^Innovation type	
• Essential vs. non-essential	A practice that provided essential care vs. non-essential care in the context of the COVID-19 pandemic
• Virtual vs. in-person	A practice that was virtual vs. in-person
Outer setting domain	Codes capturing information specific to the setting outside the facility
^a^Community characteristics	Stakeholder perception of community characteristics impacting the practice, including but not limited to socio-cultural (e.g., white-supremacy, ableism), socio-economic (e.g., social assistance, housing), socio-political (e.g., government), and/or socio-geographical (e.g., built environment) characteristics.
Patient needs and resources	Stakeholder perception of patient needs, preferences, and resources
External policies and incentives	Stakeholder perception of policies impacting the practice, e.g., the CDC’s guidance regarding the COVID-19 pandemic, VHA policy changes
Inner setting domain	Codes capturing information specific to the setting within the facility
^a^Employee needs and resources	Stakeholder perception of employee needs, preferences, and resources
Tension for change	Stakeholder perception of the level of need for the practice, including relevance or irrelevance of the practice during the COVID-19 pandemic
Compatibility	Stakeholder perception of the compatibility of the practice with existing workflows and processes
Available resources	Stakeholder perception of the resources available to support the practice, e.g., space, equipment
Process domain	Codes capturing information specific to the implementation and/or sustainment process
Executing	
• ^a^Adapting	Stakeholder perception of the extent to which the practice was or was not adapted
Engaging	
• ^a^Key stakeholders	Stakeholder perception of the extent to which employees are available and/or engaged to deliver or do the practice
^a^Outcomes domain	Codes capturing primary outcomes
Sustained: ongoing	The practice is in use and ongoing
Sustained: COVID-hold	The practice is in use but on a temporary hold due to the COVID-19 pandemic
Partially sustained	The practice is partially in use, e.g., only some components of the practice are in place
Never implemented/not re-implemented/not sustained	The practice is not in use
^a^Relationship codes	Codes (denoted by symbols) capturing relationships between constructs
|	Hindered and/or stopped
>	Facilitated and/or led to

^a^ A new inductively derived construct or domain

### Primary outcome

**Fig. 2:**
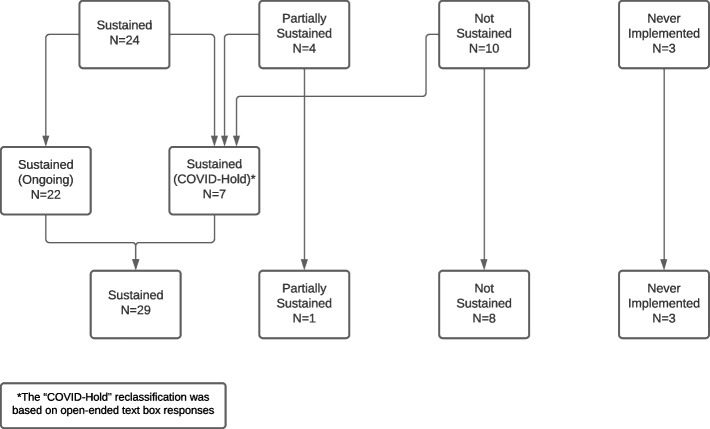
Primary outcome reclassification

We began analysis by comparing alignment of quantitative and qualitative responses to the primary outcome (i.e., “Is this practice still being used or done at your site?”) (see Table 1: Item 1). Seven facility representatives reported in the survey’s open-text box that their practice was on a temporary hold due to COVID-19 but answered the primary outcome question differently; two answered “Yes”, two answered “No”, and three answered “Partially”. As a result, the team reclassified the primary outcome of those facilities into a new category under Sustained: Temporary COVID-Hold (see Fig. 2). Following this reclassification, the number and percent of facilities in each sustainment category was calculated by cohort.

### Secondary outcomes

We calculated the number and percent of facilities for Items 2–8 (see Table 1) within each of our primary outcome categories from Item 1 (sustained, partially sustained, not sustained) (see Table 1: Item 1). We also analyzed the concordance between Items 1 (practice sustainment) and 2 (practice institutionalization) in the survey.

## Results

### Primary outcome

A representative from forty-one facilities (41/64; 64%) completed the survey in the summer of 2020 while 23 (35.9%) facility representatives were lost to follow-up; the rate of missing data was lower after the first DoE cohort. Among responding facilities, 29/41 (70.7%) facilities were sustaining their practice, 1/41 (2.4%) facilities were partially sustaining their practice, 8/41 (9.5%) facilities were not sustaining their practice, and 3/41 (7.3%) facilities had never implemented their practice (see Table 3). Sustainment rates increased across Cohorts 1–4. The CFIR constructs and inductive codes associated with primary outcome text responses are included in parentheses below; the facilitates/leads to relationship is illustrated with “>” and the hinders/stops relationship is illustrated with “|”. Please refer to Table 2 for code definitions.

**Table 3:** Practice sustainment by cohort: number (percent) of facilities

Cohort (year^a^)	Sustained^b^	Partially sustained	Not sustained	Never implemented	Total non-missing	Missing	Total
1 (2016)	4 (50.0)	0 (0.0)	3 (37.5)	1 (12.5)	8 (47.1)	9 (52.9)	17
2 (2017)	8 (66.7)	0 (0.0)	3 (25.0)	1 (8.3)	12 (71.0)	5 (29.4)	17
3 (2018)	7 (70.0)	1 (10.0)	2 (20.0)	0 (0.0)	10 (71.4)	4 (28.6)	14
4 (2019)	10 (90.9)	0 (0.0)	0 (0.0)	1 (9.1)	11 (68.8)	5 (31.3)	16
Total	29 (70.7)	1 (2.4)	8 (19.5)	3 (7.3)	41 (64.1)	23 (35.9)	64

^a^ Indicates year in which facilitated implementation support from DoE ended

^b^ This category includes 7 facilities that were on a temporary hold due to the COVID-19 pandemic

#### Sustaining facilities

Twenty-nine facilities (*N* = 41, 70.7%) were sustaining their practice (see Table 3). Of these 29 facilities, 22 (75.9%) were *ongoing* during the COVID-19 pandemic while 7 (24.1%) were on a *temporary COVID-Hold* (see Table 4). The differences between these two sustaining groups of facilities are described below.

**Table 4:** Sustained practices: ongoing vs. COVID-hold: number (percent) of facilities by cohort

Cohort (year^a^)	Ongoing	COVID-hold	Total sustained
1 (2016)	3 (75.0)	1 (25.0)	4
2 (2017)	5 (62.5)	3 (37.5)	8
3 (2018)	7 (100.0)	0 (0.0)	7
4 (2019)	7 (70.0)	3 (30.0)	10
Total	22 (75.9)	7 (24.1)	29

^a^ Indicates year in which facilitated implementation support from DoE ended

##### Sustaining facilities: ongoing

In late March 2020, the CDC issued guidance to cancel and/or reschedule “non-essential clinical activities, including elective procedures, face-to-face outpatient visits, diagnostic testing, and procedures” [32]. However, 22/29 facility representatives (75.9% of sustaining facilities) reported their practice was ongoing during this time. Many clinical practices were able to continue because they provided essential care for patients or were already virtual in nature (*Innovation Type: Essential or Virtual & Tension for Change > Sustained: Ongoing)*. In fact, the pandemic served to increase the need and therefore the spread of virtual practices:

[Virtual care] is under a huge expansion. We are just now looking at adding Nursing [virtual care] clinics […] everything [virtual care] has expanded with COVID. (Facility 4_IF02a)

In contrast, other practices were ongoing during the pandemic because they adapted the practice’s in-person events to virtual events (*External Policies & Incentives > Adapting > Sustained: Ongoing*):

We are currently orchestrating our third annual Summit (virtually because of COVID). (Facility 3_IF09c)

The other ongoing practices were designed to benefit employees or represented administrative process changes that were not impacted by the pandemic (*Employee Needs and Resources > Tension for Change > Sustained: Ongoing*):

As a [department] we use this regularly and inform our employees of their current status as we continue to perform our normal tasks and duties. (Facility 2_IF06a)

##### Sustaining facilities: COVID-hold

Although the majority of sustaining facilities were ongoing, 7/29 facility representatives (24.1% of sustaining facilities) reported they placed their practice on a temporary hold following the CDC guidance [32] (*External Policies & Incentives & Innovation Type: Non-Essential > Sustained: COVID-Hold)*. As illustrated in the following quotes, these facilities could not reasonably nor safely adapt their practice and offer it virtually (*Patient Needs & Resources | Adapting > Sustained: COVID-Hold)*:

Due to COVID-19, we are unable to use this program at this time. We are currently being encouraged to do telehealth from home. We believe this program would carry additional risks [to Veterans] should it be used by telehealth rather than face to face. (Facility 2_IF11_2)

Other practices became less applicable when very few patients were present in the hospital, e.g., practices seeking patient feedback or reporting patient metrics (*External Policies & Incentives | Tension for Change > Sustained: COVID-Hold*).

Due to the pandemic we did not have the metrics to utilize the [practice] so it was placed on hold. (Facility 1_IF05)

#### Partially sustaining facility

Only one facility (*N* = 41, 2.4%) was partially sustaining their practice (see Table 3). The respondent explained partial sustainment by noting the practice was in use “in some specialty clinics, palliative care and hospice.” (Facility 3_IF04)

#### Not sustaining facilities

Eight facilities (*N* = 41, 19.5%) were not sustaining their practice (see Table 3). Within this group, 6/8 (75%) had a previous last known status of no sustainment and 2/8 (25%) had a previous last known status of sustained or partially sustained.

##### Not sustaining facilities: facilities that were previously not sustaining

As noted in the Methods section (see Survey Development), facilities that had a last known status of no sustainment were given an introductory question to determine if they had re-implemented their practice in the interim. Six facilities (*N* = 8, 75% of the not sustaining facilities) had not re-implemented for various reasons. Two of these facilities had not re-implemented due to losing necessary staffing and not having completed re-hiring (*Engaging Key Stakeholders > Not Re-Implemented).*

[The] person that initiated this practice left and it was not followed through with new staff. (Facility 2_IF07b)

Two other facilities had not re-implemented because the practice was incompatible with patient needs, facility resources, or existing workflows (*Patient Needs & Resources & Available Resources | Compatibility > Not Re-Implemented).*

[The practice] did not meet the needs of our Veterans in [service] [and there were] issues with [the equipment] maintaining network connection [which] slowed [service] workflow. (Facility 1_IF03c)

One facility had not re-implemented after there was a policy change disallowing the practice to continue (*External Policy & Incentives > Not Re-Implemented*). There was no qualitative data explaining why the 6^th^ facility did not re-implement.

##### Not sustaining facilities: facilities that were previously sustaining

In contrast, two of the currently not sustaining facilities (*N* = 8, 25% of not sustaining facilities) had a previous status of sustained or partially sustained. Representatives from these facilities reported a lack of sustainment occurred in the previous year due to losing necessary employees (*Engaging Key Stakeholders > Not Sustained)* or finding that the practice was ineffective in their community (*Community Characteristics > Not Sustained)*.

One of our [employee] positions has been vacant since January and the other [employee] position was realigned under a specific specialty care service. (Facility 3_IF01a)

##### Not sustaining facilities: plans to re-implement practice

To better understand the fluid nature of sustainment, facilities that were not sustaining their practice were given a follow-up survey question to determine if they intended to re-implement their practice in the future. Three of the eight (38%) not sustaining facilities intended to re-implement their practice in the future (two previously not sustaining facilities and one newly not sustaining facility) (see Table 5).

**Table 5:** Plans to reimplement in not sustaining facilities: number (percent) of facilities

Response	Yes	No	Total not sustained
Number (%)	3 (37.5)	5 (62.5)	8

Two of these facilities explained that while they had lost necessary staffing, they were in process or planning to replace them to re-implement in the future (*Engaging: Key Stakeholders > Not Sustained*).

We recently hired a new Provider and are in the processes of getting her setup with [service] access/equipment. (Facility 2_IF02b)

### Secondary outcomes

The following sections describe results from secondary outcomes, which were used to contextualize the primary outcome. Of note, there was a high level of missing data for the secondary outcome questions; our branching logic omitted secondary outcome questions for facilities that did not have their practice in place, i.e., did not re-implement or sustain, including two facilities that were reclassified from Not Sustained to Sustained: COVID-Hold (see Tables 6 and 7; Footnote §). As a result, only practice effectiveness and practice institutionalization are presented below. The branching logic is illustrated in Fig. 1; reclassification of outcomes is illustrated in Fig. 2.

**Table 6:** Concordance of practice sustainment and practice institutionalization: number (percent) of facilities

	Sustained: ongoing and COVIID-hold	Partially sustained	Not sustained	Never implemented	Total non-missing
Institutionalized	23 (95.8)	1 (4.2)^a^	0 (0.0)	0 (0.0)	24
Partially Institutionalized	3 (100.0)^b^	0 (0.0)	0 (0.0)	0 (0.0)	3
Not institutionalized	1 (100.0)^c^	0 (0.0)	0 (0.0)	0 (0.0)	1
Missing	2 (15.4)^d^	0 (0.0)	8 (61.5)	3 (23.1)	13
Total	29 (70.7)	1 (2.4)	8 (19.5)	3 (7.3)	41

^a^ Practice was not in use across all services, but was institutionalized where it was in place (Facility 3_IF04)

^b^ Lack of concordance for 2/3 facilities due to reclassification of sustainment outcome (Facilities 2_IF07a, 4_IF05); the third facility did not have any qualitative data to contextualize the responses Facility 4_IF09c)

^c^ Practice was sustained but had not spread (Facility 4_IF02a)

^d^ Branching logic omitted the institutionalization question for facilities that never implemented/did not sustain, including for two facilities that were reclassified from not sustained to sustained: COVID-hold

**Table 7:** Practice effectiveness: number (percent) of facilities

	Sustained: ongoing and COVID-hold	Partially sustained	Not sustained	Never implemented	Total non-missing
Yes	23 (79.3)	0 (0.0)	0 (0.0)	0 (0.0)	23
Partially	1 (3.4)^a^	1 (100.0)^b^	0 (0.0)	0 (0.0)	2
No	2 (6.9)^c^	0 (0.0)	0 (0.0)	0 (0.0)	2
Missing	3 (10.3)^d^	0 (0.0)	8 (100.0)	3 (100.0)	14
Total	29	1	8	3	41

^a^ This facility received feedback from employees and was considering adapting the practice to make it more effective at their facility (Facility 2_IF07a)

^b^ This facility did not provide any qualitative data on this question (Facility 3_IF04)

^c^ One facility found the practice to be ineffective (Facility 4_IF09c) and the other was not tracking (Facility 4_IF02a)

^d^ Branching logic omitted the effectiveness question for facilities that never implemented/did not sustain, including for two facilities that were reclassified from not sustained to sustained: COVID-hold. The final facility in this category was also a COVID-hold facility

#### Practice institutionalization

Overall, there was a high level of concordance (96%) between sustainment and institutionalization outcomes (see Table 6). In addition, two of the three facility representatives that reported partial institutionalization also reported partial sustainment, reflecting initial concordance; however, those two facilities were reclassified from partially sustained to sustained: COVID-hold during analysis (see Table 6, Foot Note † and Fig. 2).

Though less frequent, three facilities had discordant sustainment and institutionalization outcomes. The qualitative data from the survey provided additional context to explain some of the reasons for this discordance. For example, the facility representative that reported partial sustainment (see above) reported the practice was institutionalized where the practice was in use, but it was only in use “in some specialty clinics, palliative care and hospice” (see Table 6, Footnote *). Another facility representative reported the practice was sustained but not institutionalized; though the practice was in use where it was initially implemented, they stated “we want it to expand” (Facility 4_IF02a) (see Table 6, Footnote ‡).

#### Practice effectiveness

Of the 29 facilities sustaining their practice, 23 representatives (79.3%) reported the practice was demonstrating effectiveness (see Table 7). They reported using a variety of measures appropriate to their practices to track effectiveness, including patient-level (e.g., clinical measures, satisfaction rates), employee-level (e.g., turnover rates), and system-level metrics (e.g., time and cost savings). For example, one facility representative reported their practice led to a “decrease[d] LOS [length of stay for patients in the hospital] and higher patient satisfaction scores.” (Facility 4_IF07b).

One representative (*N* = 29, 3.4%) reported the practice was partially demonstrating effectiveness, stating they had received feedback from employees that the practice was not fully meeting their needs and they were considering adapting the practice to make it more effective at their facility (Facility 2_IF07a) (see Table 7, Footnote *). Two representatives (*N* = 29, 6.9%) reported the practice was not demonstrating effectiveness; one representative reported the practice “was found to be ineffective with our non-traditional patient population” and they were “transitioning to new presentation and process,” (Facility 4_IF09c) while the other reported they were “not tracking” and therefore were not able to demonstrate effectiveness (Facility 4_IF02a) (see Table 7, Footnote ‡).

## Discussion

With the growing attention on sustainment of EIPs, there is a need for clarity in defining and measuring sustainability versus sustainment. Given that funding often ends before longer-term sustainment can be assessed, it is important for researchers to develop pragmatic sustainment measures that can be used when there are fewer resources and incentives for participants.

As part of an ongoing evaluation of the VHA DoE, we developed and piloted a pragmatic survey to assess ongoing sustainment across diverse practices. Based on the relatively high response rate (over 60%) and logical responses provided, we can discern several pragmatic features: it was short, easy to understand, and applicable across a wide range of practices [21, 31].

Survey results indicated a high rate (over 70%) of practice sustainment among responding facilities, which suggests that the VHA DoE is a promising large-scale model of diffusion. Sustainment rates increased across Cohorts 1–4, with later cohorts reporting higher rates of sustainment than earlier cohorts. Ongoing enhancements made to the VHA DoE processes over time (e.g., refining methods to select Promising Practices, better preparing facilities for implementation) may have helped improve sustainment rates over time. It’s also possible lower rates in Cohorts 1–2 (2016 and 2017) highlight challenges to sustainment over longer periods. However, only two additional facilities discontinued their practice in the year prior to the survey and these were part of Cohort 3 (2018). Future sustainment surveys with these and new cohorts will help build understanding about changes over time and factors that help or hinder ongoing sustainment. Our ability to continue following these practices is a unique strength of this evaluation.

### Lessons learned

#### One: multiple-choice responses

There were several important lessons learned that will improve our ongoing evaluation efforts and subsequent surveys. *First*, our primary measure failed to capture nuance in the data related to practices being temporarily on hold. Our survey was piloted during the COVID-19 pandemic, during which the CDC issued guidance to cancel and/or reschedule non-essential in-person healthcare. As a result, several respondents used the open-text boxes to explain that their practice was in place but on hold during the pandemic, and that they planned to resume operations in the future. However, facility representatives were not consistent in how they answered the primary question; responses ranged from sustained to partially sustained to not sustained. Based on content analysis of open-text explanations, we systematically reclassified these responses as sustained: COVID-hold “to mitigate survey bias and ensure consistency” [20] (see Fig. 2). Though temporary holds were common in our evaluation due to the pandemic, EIPs may be paused for a variety of reasons that do not necessarily indicate discontinuation and lack of sustainment. For example, two facility representatives reported their practice was not sustained because they lost employees, but they were in the process of re-hiring; in effect, though the reason was different, these practices were on hold similar to practices paused by the pandemic. It is important to note that turnover and gaps in staffing aligns with a key finding from our earlier work: when implementation and sustainment are achieved via the efforts of a single key employee, it is impossible to reliably sustain the practice when that person leaves or simply takes vacation [28].

In the future, we will add responses to capture whether the practice has been discontinued permanently or is temporarily not in use/not in place. In addition to better fitting the data, this refinement allows the measure to be used at any time point from initial adoption to sustainment; although adoption, implementation, and sustainment are defined differently based on the measurement point, they all assess whether the innovation is being used or delivered [5]. This refinement further shortens the survey by eliminating the need for a follow-up question about re-implementation of the practice.

#### Two: sustainment determinants and outcomes

*Second*, the sustainment literature often conflates sustainment *determinants* with sustainment *outcomes*. Table 1 lists measures conceptualized as outcomes in the literature that were included in our survey. However, if a facility representative reported the practice was not in use (our primary outcome), many of the secondary outcomes were not applicable to that facility. For example, if a practice was not in use, asking whether the practice was demonstrating effectiveness would be illogical; continued effectiveness is a *determinant* to successful sustainment, not an *outcome*. Since we did not include secondary outcomes for those who reported they were not sustaining their practice, there was a high rate of missing data for these items by design. Future versions of the survey will reconceptualize Items 3–7 in Table 1 as sustainment determinants which aligns with the SMSS but with fewer items [23].

Item 2 (Practice Institutionalization) was correlated with our primary sustainment outcome (Item 1). Goodman and Steckler define institutionalization as the “long-term viability and integration of a new program within an organization” [35]. Institutionalization is conceptualized as a deeper, more mature form of sustainment; where the practice is fully routinized and embedded into clinical practice, beyond just relying on the effort of a single person [36]. Basic sustainment (whether a practice is in use) would be a prerequisite for practice institutionalization. Finally, Item 9 (Practice Spread/Diffusion) will be conceptualized as a diffusion outcome. Rogers defines diffusion as “the process through which an innovation […] spreads via certain communication channels over time” [37] within and across organizations [38]. Survey respondents may report sustainment within their own setting with or without diffusion to additional sites. A key goal for the DoE is to widely diffuse effective practices across clinical settings within and outside VHA.

#### Three: open-text reponses

*Third*, we used “please explain” as a prompt for our open-text boxes to provide respondents with an opportunity to contextualize their experiences. However, the information they provided often focused on the rationale for the response rather than barriers and facilitators that led to their reported outcome. For example, when a facility representative reported a practice was sustained, they provided a rationale for their answer (e.g., all core components were in place) vs. a description of facilitators that allowed them to sustain their practice (e.g., continued funding). Changing this prompt to “Why?” and reconceptualizing Items 3–7 of our survey as sustainment determinants (see above) will more directly assess relevant barriers and facilitators.

#### Four: sustainability

*Fourth*, we will add a sustainability question (i.e., elicit prospects for continued sustainment) to the survey for all respondents. Although we asked not sustaining facilities a prospective question about plans to re-implement, we did not ask sustaining facilities a prospective question about continued sustainment. Our previous work indicated that predictions of sustainment were relatively accurate [28]. Sustainment is dynamic and may ebb and flow over time; those working most closely with the practice are best positioned to assess prospects for future sustainment as well as anticipated barriers. Low ratings of sustainability could provide an opportunity for early interventions to stave off future failure to sustain.

### Pragmatic sustainment surveys and future directions

It is important to note that following piloting of our survey, Moullin et al. published the Provider REport of Sustainment Scale (PRESS); it contains three Likert Scale items: 1. Staff use [EIP] as much as possible when appropriate; 2. Staff continue to use [EIP] throughout changing circumstances; 3. [EIP] is a routine part of our practice [39]. To our knowledge, the PRESS is the first validated pragmatic sustainment instrument and addresses key issues highlighted by Moullin et al. in their previous narrative review [21] (see Background: Limitations to current instruments). Although the PRESS is an excellent instrument, we believe that our updated survey tool will offer unique strengths.

First, our measure is intended to be used annually; in order to maintain an up-to-date participant list, we need to know which practices are discontinued permanently vs. temporarily on hold. As a result, the multiple response options in our survey will allow us to capture important nuance in the data (see Lessons learned one). Second, a brief section on determinants will allow us to understand the barriers and facilitators that explain the sustainment status (see Lessons learned two). Third, the inclusion of a sustainability item (i.e., the likelihood of future sustainment) offers DoE leadership earlier opportunities to intervene when facility representatives predict their practice may fail to be sustained (see Lessons learned three). Finally, the inclusion of open-text responses contributes to measurement accuracy, provides additional contextual information, and facilitates uncovering unexpected themes [20], all of which are urgently needed in an increasingly uncertain world (see Lessons learned four). If additional piloting shows the survey is useful, we plan to assess the reliability and validity of this tool for broader use in the field.

## Limitations

Although we tried to limit bias, this is a real-world quality improvement project, and there are several limitations that may have skewed our results: (1) the use of self-report, potential social desirability, and lack of fidelity assessment; 2) the size of our sample; and (3) the rate of missing data. Regarding the first limitation: Although self-report may be less objective than in-person observation, it is commonly used to assess sustainment (e.g., both the SMSS [23] and PRESS [39] rely on self-report), because it is more pragmatic and feasible [39]. Given travel and funding limitations, self-report was the only option in our project. To limit social desirability bias, we informed participants that we were not part of the DoE team, that the survey was completely voluntary, and that no one outside of the evaluation team would have access to their data. In addition, our survey was unable to include innovation-specific components of fidelity due to the diverse nature of DoE practices. However, the generic nature of the tool is one of its strengths; although this work has been limited to the VHA, it was used across a wide portfolio of diverse practices. Thus, the tool may be useful in other healthcare systems. Regarding the second limitation: Although the sample size was small, all representatives involved in implementation were invited to complete the survey. However, as additional cohorts participate in the DoE, the sample size will increase, allowing us to pilot the survey with additional facilities and practices. Regarding the third limitation: Although the rate of missing data was 40%, it generally decreased with each new cohort; this may be a function of shorter time periods elapsed since initial implementation; however, we plan to continue including non-responding facilities in future surveys until they have been lost to follow-up for 3 years.

## Conclusions

We provide further clarity for concepts of sustainability and sustainment and how each is measured. The initial development and piloting of our pragmatic survey allowed us to assess the ongoing sustainment of DoE practices, demonstrating that the DoE is a promising large-scale model of diffusion. If additional piloting illustrates the survey tool is useful, we plan to assess its reliability and validity for broader use in the field; given our survey was used with a diverse portfolio of practices, it may serve as a useful survey tool for other evaluation efforts.

## Supplementary Information

**Additional file 1.** Cohort 1 – 4 Practice Descriptions. This file provides descriptions of each of the Promising Practices that were included in this evaluation.

**Additional file 2.** Sustainment Survey. This file is the survey that was piloted with participants in this evaluation.

## Data Availability

The datasets generated and/or analyzed during the current evaluation are not available due to participant privacy but may be available from the corresponding author on reasonable request.

## Abbreviations

CFIR: Consolidated Framework for Implementation Research
DoE: Diffusion of Excellence
EIP: Evidence-informed practice
VHA: Veterans Health Administration
