# Supplementary material for: Sustainment of diverse evidence-informed practices disseminated in the Veterans Health Administration (VHA): initial development and piloting of a pragmatic survey tool
Source: Implement Sci Commun. 2023 Jan 16;4:6. doi: 10.1186/s43058-022-00386-z (PMC9842210; doi:10.1186/s43058-022-00386-z)
Supplement: Supplementary file 2 — Additional file 2. Sustainment Survey. This file is the survey that was piloted with participants in this evaluation. [file 43058_2022_386_MOESM2_ESM.pdf]

Survey

Please complete the survey below about the Diffusion of Excellence, the current status of the Gold Status Practice titled [gsp_name], and the COVID-19 pandemic.

Thank you!

Practice Status: This section asks questions about the status of the practice titled [gsp_name].

Based on our last contact with your site, it is our understanding that your site had not fully sustained this practice: Did your site reimplement this practice?
◯ Yes
◯ No
◯ Partially

Please Explain: __________________________________

Are there future plans to reimplement this practice?
◯ Yes
◯ No

Please Explain: __________________________________

Based on our last contact with your site, it is our understanding that your site had not fully completed implementation of this practice: Did your site complete implementation of this practice?
◯ Yes
◯ No
◯ Partially

Please Explain: __________________________________

Are there future plans to complete implementation of this practice?
◯ Yes
◯ No

Please Explain: __________________________________

Is this practice still being used or done at your site?
◯ Yes
◯ No
◯ Partially

Please Explain: __________________________________

Are there future plans to reimplement this practice?
◯ Yes
◯ No

Please Explain: __________________________________

Is this practice considered routine, usual practice? (i.e., practice is nearly always used or done when appropriate by all individuals involved)

◯ Yes
◯ No
◯ Partially

Please Explain: ______________________________

On a scale of 1 to 5, where 1 is strongly disagree and 5 is strongly agree, please rate the following statements:

	1 Strongly Disagree	2	3	4	5 Strongly Agree	N/A
This practice has support and commitment from facility leadership	◯	◯	◯	◯	◯	◯
This practice has sufficient funding	◯	◯	◯	◯	◯	◯
This practice has sufficient resources (e.g., space, equipment)	◯	◯	◯	◯	◯	◯
This practice has a Champion (leader) at your site	◯	◯	◯	◯	◯	◯
This practice has sufficient staffing	◯	◯	◯	◯	◯	◯
This practice has priority at your site	◯	◯	◯	◯	◯	◯
This practice has support and buy-in from key outside community entities	◯	◯	◯	◯	◯	◯

Please Explain: ______________________________

Is this practice demonstrating effectiveness at your site? ◯ Yes ◯ No ◯ Partially

What indication do you use to decide its effectiveness? ______________________________

Please Explain: ______________________________

Have there been any changes or adaptations to this practice? ◯ Yes ◯ No

Please Explain: ______________________________

Has this practice spread to other places outside your site? ◯ Yes ◯ No

Please Explain: __________________________________

Has this practice spread to other units or places in your site? ◯ Yes ◯ No

Please Explain: __________________________________

COVID-19: This section asks questions about the Diffusion of Excellence, the practice titled [gsp_name], and the COVID-19 pandemic.

What impact did the COVID-19 pandemic have on this practice? __________________________________

How has your involvement in the Diffusion of Excellence (e.g., skills developed, connections with people) affected your ability to respond to COVID-19?

Please consider your ability to carry out activities directly and indirectly related to COVID-19 care.

Quality Improvement or leadership activities directly related to COVID-19 care (e.g., screening, testing, treatment, or surveillance of COVID-19): __________________________________

Quality Improvement or leadership activities indirectly related to COVID-19 (e.g., rearranging care processes, use of telehealth, addressing overall well-being of Veterans and staff): __________________________________

Other professional or personal responses to the COVID-19 pandemic: __________________________________

Based on your VA experience in response to the COVID-19 pandemic, is there anything else you want to communicate to the Diffusion of Excellence or would like the Diffusion of Excellence to communicate to others in the VA? __________________________________

This may include successes, challenges, or anything else.
